# E2F1-induced autocrine IL-6 inflammatory loop mediates cancer-immune crosstalk that predicts T cell phenotype switching and therapeutic responsiveness

**DOI:** 10.3389/fimmu.2024.1470368

**Published:** 2024-10-31

**Authors:** Alf Spitschak, Prabir Dhar, Krishna P. Singh, Rosaely Casalegno Garduño, Shailendra K. Gupta, Julio Vera, Luca Musella, Nico Murr, Anja Stoll, Brigitte M. Pützer

**Affiliations:** ^1^ Institute of Experimental Gene Therapy and Cancer Research, Rostock University Medical Center, Rostock, Germany; ^2^ Department of Systems Biology and Bioinformatics, University of Rostock, Rostock, Germany; ^3^ Department of Biomedical Engineering & Bioinformatics, Chhattisgarh Swami Vivekananda Technical University, Bhilai, Chhattisgarh, India; ^4^ Laboratory of Systems Tumor Immunology, Department of Dermatology, Uniklinikum Erlangen and Friedrich-Alexander Universität Erlangen-Nürnberg (FAU), Erlangen, Germany; ^5^ Department Life, Light & Matter, University of Rostock, Rostock, Germany

**Keywords:** E2F1-STAT3/IL-6 network, melanoma secretome, tumor microenvironment, immunomodulation, CD4 +/CD8 + T cells, Th2-Th1 shift, cancer metastasis, mathematical modeling

## Abstract

Melanoma is a metastatic, drug-refractory cancer with the ability to evade immunosurveillance. Cancer immune evasion involves interaction between tumor intrinsic properties and the microenvironment. The transcription factor E2F1 is a key driver of tumor evolution and metastasis. To explore E2F1’s role in immune regulation in presence of aggressive melanoma cells, we established a coculture system and utilized transcriptome and cytokine arrays combined with bioinformatics and structural modeling. We identified an E2F1-dependent gene regulatory network with IL6 as a central hub. E2F1-induced IL-6 secretion unleashes an autocrine inflammatory feedback loop driving invasiveness and epithelial-to-mesenchymal transition. IL-6-activated STAT3 physically interacts with E2F1 and cooperatively enhances IL-6 expression by binding to an E2F1-STAT3-responsive promoter element. The E2F1-STAT3/IL-6 axis strongly modulates the immune niche and generates a crosstalk with CD4^+^ cells resulting in transcriptional changes of immunoregulatory genes in melanoma and immune cells that is indicative of an inflammatory and immunosuppressive environment. Clinical data from TCGA demonstrated that elevated E2F1, STAT3, and IL-6 correlate with infiltration of Th2, while simultaneously blocking Th1 in primary and metastatic melanomas. Strikingly, E2F1 depletion reduces the secretion of typical type-2 cytokines thereby launching a Th2-to-Th1 phenotype shift towards an antitumor immune response. The impact of activated E2F1-STAT3/IL-6 axis on melanoma-immune cell communication and its prognostic/therapeutic value was validated by mathematical modeling. This study addresses important molecular aspects of the tumor-associated microenvironment in modulating immune responses, and will contribute significantly to the improvement of future cancer therapies.

## 1 Introduction

Tumor metastasis is the leading cause of mortality in cancer patients. At each step, metastasis involves a complex cascade of events that includes a tight bidirectional interaction between cancer cells and immune system (1). As a tumor evolves and during its progression, neoplastic cells adopt a variety of intrinsic traits driven by genetic and epigenetic aberrations, deregulated signaling or altered metabolism, and hijack immune cells to orchestrate an inflammatory immunosuppressive tumor microenvironment (TME) (2). Depending on the tumor type and stage, different immune cell populations such as T and B lymphocytes, macrophages, dendritic cells (DCs), natural killer cells (NK), and neutrophils contribute to the TME. They exhibit considerable diversity and plasticity, and respond to environmental signals by acquiring distinct functional phenotypes that either inhibit or promote metastasis (1). The mechanisms by which cancer cells can evade an immune response are manifold comprising the induction of immune cell exhaustion, recruitment and expansion of immunosuppressive cells, and the production of immunosuppressive cytokines and factors like immune checkpoint molecules that impair cell functions, shifting the immune response towards immunosuppression (3). This reciprocal crosstalk between cancer and immune cells not only supports immune escape and metastatic spread, but also the efficiency of immunotherapies that render the mediators or mechanisms responsible for immune reprogramming attractive targets for drug development. Potential target structures for treatment are intracellular variables such as transcription factors (TF) and their interactome, coregulatory molecules, ligand-receptor interaction, exosomes or secretomes. Thus, to overcome current therapeutic limitations a better understanding of the intricate communication between cancer cell characteristics and the tumor immunological microenvironment (TIME) may provide a foundation for the development of advanced personalized immune intervention therapies, which is best demonstrated by the partial clinical success of immune checkpoint inhibitors (ICI).

Melanoma is a skin cancer with increasing prevalence and infaust once it reaches a metastatic stage. In general, it is considered an immunogenic tumor that expresses several tumor-specific as well as tumor-associated antigens as a result of chromosomal rearrangements or genetic polymorphisms (4). Tumor-specific T cell responses have been detected in a significant portion of patients (5). Moreover, adoptive transfer of tumor-specific T cells can mediate durable responses in patients with metastatic melanoma. Recently, ICI have revolutionized the treatment of advanced melanoma, achieving tumor regression and long-lasting cancer control in nearly 50% of patients (6). These high response rates underscore the immunogenicity of this tumor entity and suggest that in most patients the antitumor immune response is impaired and can only be restored in a subpopulation by currently available immunomodulators (7). Besides an increased production of inhibitory molecules such as PD-L1, the ligand for PD-1 receptor and target of ICI, melanoma can block immune cell activation by a plethora of mechanisms (8). Hyperactivation of tumor-intrinsic pathways like BRAF-MAPK or WNT/β-catenin signaling, for instance, has been shown to mediate immune cell exclusion (9, 10). Moreover, recruitment of immunosuppressive cells such as regulatory CD4^+^ T cells (Tregs), CD4^+^ T helper 2 (Th2) and B cells, tumor-associated macrophages (TAMs) and myeloid-derived suppressor cells (MDSCs) can inhibit antitumor T cell responses and promote tumor proliferation, metastasis, and angiogenesis (11). Another key influential factor to regulate the interaction between melanoma and recruited immune cells in the TME is the secretion of cancer-derived chemokines or cytokines (12) and negative modulators of immune cell activation, including adenosine, vascular endothelial growth factor (VEGF), and indolamine-2,3-dioxygenase (4). Melanoma progression is also correlated with the presence of Tregs and Th2 within the tumor and that their recruitment by cancer cells helps them to avoid the immune response, mainly through secreted tumor growth factor β (TGF-β) and interleukins (ILs) (11, 13).

A key epigenetic player in melanoma pathology is the E2F1 transcription factor. Beyond its physiological role as regulator of the cell cycle, metabolism, or tumor surveillance factor via apoptosis induction in response to DNA damage, abundantly high E2F1 expression stimulates metastatic transformation in melanoma and several other cancers (14–16). This TF orchestrates metastatic spread by promoting epithelial-to-mesenchymal transition (EMT), neoangiogenesis, extravasation, and genomic instability, predicting disease exacerbation, and poor patient outcome (17). Ablation of E2F1 restores E-cadherin and induces death of metastatic melanoma cells resistant to BRAF inhibitor expression (14). Through integration of logic-based network modeling and gene expression profiling of cancer cell lines from E2F1-driven tumors and patient cohorts with aggressive cancer, tumor type-specific receptor signatures associated with EMT were found, in which the combined activity of high E2F1, TGFBR1, and FGFR1 triggers the most invasive phenotype (15). Previous studies have shown that the aggressive activity of E2F1 in cancer cells is largely dependent on the spatiotemporal availability of transcriptional coregulators that can enhance its transcriptional programs through formation of protein-protein interaction (PPI) complexes to favor the expression of genes that support a metastasis-prone TME (17–19). A good example for cooperativity associated with changes in the tumor immune contexture is the complex between E2F1 and metastasis-associated protein 1 (MTA1). This complex potentiates cell motility and pulmonary metastasis *in vivo* via increased expression of hyaluronan synthase 2 (HAS2) and hyaluronic acid (HA) production, which in turn activates infiltration of M2-type TAMs in the TME. Disruption of the E2F1-MTA1 complex efficiently impairs the establishment of a prometastatic TME, which is achieved by reducing the HAS2/HA production (19). Considering the engagement of immune cells with cancer cells, a crucial mechanism for tumor progression along the metastatic cascade is a strong case for an immunomodulatory potential of this TF. The exact role of E2F1 signaling in the immune niche, the extent it regulates antitumor immunity, and consequently, the underlying mechanisms are widely unknown.

Here, we demonstrate that E2F1 is of outstanding importance for melanoma-immune cell communication. We investigated the immunomodulatory properties of the E2F1-induced melanoma secretome in an indirect coculture system with healthy donor-derived CD4^+^ and CD8^+^ T cells. Using transcriptomic profiling, bioinformatic analyses, and structural modeling, we found that E2F1 orchestrates the tumor niche by activating an IL6-based gene regulatory network (GRN) and a highly inflammatory secretome. IL-6 expression, driven by a newly identified interaction between endogenous E2F1 and active STAT3, leads to massive cytokine secretion via a positive feedback loop, both in melanoma and CD4^+^ T cells. Monitoring T cell activity revealed changes in the transcriptional landscape and secretome of CD4^+^ T cells that are indicative of a Th2-polarized immune response, which could be shifted toward T helper 1 (Th1) by transcriptional perturbation of E2F1. Consistently, upregulation of E2F1 and IL-6 positively correlates with tumor infiltration of immunosuppressive Th2 cells in melanoma patients. Mathematical modeling of the discovered E2F1-STAT3/IL-6 axis supported by patient data provides a powerful tool for the rational design of personalized next-generation immunotherapeutics.

## 2 Materials and methods

### 2.1 Melanoma cell culture and stable transduction

The melanoma cell lines SK-Mel-29, Mel888, SK-Mel-103, SK-Mel-147, and C8161 were maintained as described (20). All cell lines were tested negative for mycoplasma. The procedure of generating lentiviral vectors expressing shRNA against E2F1 (clone ID: TRCN253) or scrambled control shRNA (SHC002) were previously described (19). Lentiviral transduction of the cell lines was performed for 72 h and stable cell clones were generated by puromycin selection (2 µg/ml).

### 2.2 T cell culture

Human peripheral blood mononuclear cells (PBMCs) were isolated from buffy coats of healthy donors using Pancoll separating solution (#P04-601000, PAN-Biotech) and cryopreserved according to standard protocols. CD4^+^ and CD8^+^ T cells were selected from PBMCs by using either CD4 (#130-045-101) or CD8 (#130-045-201) microbeads (Miltenyi Biotec). Selected T cells were kept activated for 72 h with a low dosage of 0.25 µg/ml phytohemagglutinin (PHA) in RPMI 1640 supplemented with 10% fetal bovine serum, 10 mM HEPES, 1% Penicillin/Streptomycin, 1 µg/ml Amphotericin B, 2 mM L-glutamine, and 20 U/ml IL2. All donors had given their written consent.

### 2.3 Indirect coculture of melanoma with either CD4^+^ or CD8^+^ T cells

Melanoma cell lines expressing shctrl or shE2F1 were cocultured with either CD4^+^ or CD8^+^ T cells in trans-well plates (#353502, Corning). Briefly, melanoma cells (1×10^5^ cells/well) were seeded and allowed to rest for 24 h. Either CD4^+^ or CD8^+^ T cells were added into the inserts (#353102, Corning) of the trans-well plates and cultivated further in a 5% CO_2_ humidified atmosphere for 72 h in the presence of 20 U/ml IL2 and 0.25 µg/ml PHA to support T cell activation and proliferation (21).

### 2.4 Flow cytometry

T cells were harvested after 72 h of culture and stained with Zombie Green™ Fixable Viability Kit (#423111, BioLegend) according to manufacturer’s instructions followed by incubation with FITC-coupled anti-human CD3 (#300306), PE-Cy7-coupled anti-human CD4 (#300512), PE-coupled anti-human CD8 (#344706), and PerCP-coupled anti-human CD279 (PD-1, #329938) antibodies (BioLegend).

### 2.5 ELISA

IL-6 (#430515, BioLegend) and interferon-γ (IFN-γ; #430105, BioLegend) concentrations were determined in the supernatants from T cell cultures by ELISA according to manufacturers´ instruction.

### 2.6 Human cytokine array

To measure secreted proteins Proteome Profiler Human Cytokine Array Kit was used according to manufacturer’s instruction (#ARY005B, R&D Systems). Briefly, cell culture supernatants from mono- or cocultured melanoma and T cells were collected to simultaneously detect 36 factors including cytokines, chemokines and acute phase proteins. Captured proteins were visualized with ECL (#32106, Thermo Fisher Scientific) and imaged with ChemiDoc™ Touch Imaging System (Bio-Rad). Spot densities were quantified using Image lab software v6.0 (#1709690, Bio-Rad) and normalized against the internal standards.

### 2.7 Cytokine and drug treatment

Cells were treated with 30 ng/ml of human recombinant IL-6 (#130-095-352, Miltenyi Biotec) and/or 30 ng/ml neutralizing IL-6 antibody (#mabg-hIL6-3, Invitrogen).

### 2.8 Immunoprecipitation and Western blot

Immunoprecipitation (IP) was performed as previously described (18). Briefly, 0.5 mg of purified cell lysate were precipitated with 0.2 µg E2F1 antibody (#3742, Cell Signaling) or unconjugated normal rabbit IgG (#2729, Cell Signaling). Protein G Sepharose beads (#17061801, Cytiva) were added to bind and purify the protein-protein complexes. After extensive washing, samples were boiled in SDS sample buffer at 95°C for 10 min to separate the proteins which were subsequently fractionated in SDS-PAGE and detected by Western blots as described (20). Antibodies were used against E2F1 (#3742), IL-6 (D3K2N, #12153), STAT3 (124H6, #9139), Phospho-STAT3 (Tyr705, D3A7, #9145; all from Cell Signaling Technology), actin (AC-15, #A1978, Sigma-Aldrich), and vimentin (V9, sc-6260, Santa Cruz Biotechnology).

### 2.9 RNA isolation and qPCR

RNA isolation and qPCR was performed as described (19). Total RNA from melanoma and T cells was extracted using the NucleoSpin^®^ RNA kit (Macherey-Nagel) and reverse-transcribed using First Strand cDNA Synthesis Kit (Thermo Fisher Scientific). For qPCR, cDNA was added to iTaq™ Universal SYBR Green Supermix (Bio-Rad) and analyzed using CFX96 Touch™ RealTime PCR Detection System (Bio-Rad). Relative gene expression was calculated by comparative CT method and normalized to GAPDH or β-actin. The following primers were used (5’-3’): E2F1_F, TCACGCTATGAGACCTCACT; E2F1_R, CTCAAGGACGTTGGTGATGT; IL6_F, ATGAGGAGACTTGCCTGGTG; IL6_R, CTGGCATTTGTGGTTGGGTC; β-actin_F, CGGGAAATCGTGCGTGACATTA; β-actin_R, ACCGCTCATTGCCAATGGTGAT; GAPDH_F, ATCGTGGAAGGACTCATGACCACA; GAPDH_R, AAGGCCATGCCA GTGAGCTTC.

### 2.10 Chromatin immunoprecipitation

Chromatin immunoprecipitation (ChIP) assay was carried out as described (19). The immunoprecipitated DNA fragments were amplified by PCR using the primers for specific E2F1 binding sites (BS; 5’-3’): ChIPA-F: TCCCCCTAGTTGTGTCTTGC; ChIPA-R: ATCTTTGTTGGAGGGTGAGGG; ChIPB-F: AGGACTGGAGATGTCTGAGG; ChIPB-R: GGGCTAAGGATTTCCTGCAC.

### 2.11 Promoter cloning and luciferase reporter assay

ChIP-seq cluster for E2F1 and STAT3 on the IL6 promoter were received from UCSC genome browser (RRID: SCR_005780; https://genome.ucsc.edu/) and JASPAR for specific BS (https://jaspar.elixir.no/; relative profile score threshold: 85%). The IL6 promoter luciferase construct pGL4.10 [luc2]-IL6 was generated by amplifying the 969 bp fragment from human genomic DNA using forward: 5′-CCTGGAGACGCCTTGAAGTA-3′ and reverse: 5′-GGAATCTTCTCCTGGGGGTA-3′ primers, cloned into pcDNA™3.1/V5-His TOPO™ TA (#10575383, Thermo Fisher Scientific). Subsequently, the insert was excised with *KpnI* and *XbaI*, incubated with DNA Polymerase I, treated with the large Klenow fragment M0210 (NEB), cloned into the *EcoRV* site of pGL4.10[luc2] from Promega, and verified by sequencing. E2F1 and both its mutant expression plasmids (E132 and ΔTA) have been described (18). Transient transfection was performed using TurboFect™ (Thermo Fisher Scientific). SK-Mel-147 (ctrl/E2F1-KD) and C8161 (ctrl/E2F1-KD) cells were transiently transfected with 0.4 µg pGL4.10 [luc2]-IL6, or 0.2 µg of either E2F1 or both mutants together with 0.1 µg pGL4.75 [hRLuc/CMV], and incubated for 24 h. Cells treated with 5 µM Stattic (#HY-13818, MedChemExpress) and incubated for 3 h, followed by cell harvest and lysis. Luciferase activity was measured using the Dual-Luciferase Reporter Assay System (Promega).

### 2.12 Invasion and cell viability assays

Matrigel and cell viability assays were conducted as described (19). Melanoma cell lines (ctrl/E2F1-KD) were seeded at a density of 2.5x10^3^ cells/well in a total volume of 100 µl in 96-well plates. For XTT assay (#4891-025-K, R&D Systems), supernatants were removed and the cells incubated for 2 h with 150 µl of TACS XTT labeling mixture (100 µl fresh growth medium, 50 µl XTT, reagent and 1 µl XTT activator). Afterwards, the absorbance was measured at 490 nm using a microplate reader. Cell proliferation kinetics were measured every 24 h for 3-4 days.

### 2.13 Molecular docking

To elucidate the mode of interaction and binding affinity of STAT3 and E2F1 with the IL6 promoter, we designed a 3D model of a 34 bp long fragment from the IL6 promoter sequence which includes the STAT3 and E2F1 BS using Biovia Discovery Studio 2022. The 3D model of E2F1 was received from previous work (17). For molecular docking, we selected first the DNA binding domain of E2F1 and the indicated E2F1 binding motif followed by a two-step molecular docking analysis of STAT3 and E2F1 with the promoter fragment. The 3D structure of STAT3 was acquired RCSB Protein Data Bank (RCSB PDB) using ID: 6QHD (22). Interaction analyses of the complex were performed using the HDOCK server (http://hdock.phys.hust.edu.cn) (23), which integrates template-based modeling and *ab initio* docking to facilitate investigations into protein-protein and protein-DNA/RNA interactions. Molecular dynamics simulations of the protein-protein and protein-DNA complexes were accomplished with the cascade protocol in BIOVIA Discovery Studio (DS2022) and subjected to molecular dynamics simulation in an implicit solvent environment focusing on diverse electrostatic interactions. The Standard Dynamics Cascade protocol of DS2022 was employed and the complex underwent an initial minimization phase of 5,000 steps using the steepest descent algorithm, followed by an additional 10,000 steps employing the Adopted Basis NR method with the CHARMm force field (24). After minimization, a heating phase incrementally raised the initial temperature from 50 K to 300 K in 50 ps intervals. Subsequently, a 50 ps equilibration step was performed, configuring the adjusted velocity frequency at 50 for both heating and equilibration phases. Following the preparation phases, a production run of 15 ns was conducted within an NVT assembly (maintaining normal volume and temperature) at a constant temperature of 300 K, with results saved at intervals of 0.02 ns. Each simulation run involved the analysis of trajectories comprising 7,500 conformations. Various properties, including root-mean-square deviation, and the bond information, were examined using the Analyze Trajectory Protocol of DS2022.

### 2.14 Microarrays and GSEA

RNA was isolated from melanoma cell lines (ctrl/E2F1-KD) and from CD4^+^ and CD8^+^ T cells. Equal amounts were subjected to Clariom™ D Assay (Thermo Fisher Scientific) and each analysis was done in triplicate. Background-corrected signal intensities were determined, processed and normalized using the Transcriptome Analysis Console (TAC 4.0, Affymetrix) and the SST-RMA algorithm. Significantly, differentially regulated targets (p value < 0.05, |Δ| ≥ 1-fold) in test samples vs. corresponding controls were determined. For gene set enrichment analysis (GSEA) differential expressed genes (DEGs; P<0.05, |Δ|>1.5-fold cut-off) were analyzed with GSEA software (v.4.3.3) using the Hallmark gene set (25–27).

### 2.15 Functional network and gene ontology analysis

To extracted the list of potential E2F1 target genes the TRANSFAC database (https://genexplain.com/transfac/) was used considering sequence segments of 10,000 bp upstream of annotated genes. The ImmuneSigDB gene signature (subset of C7) was downloaded from MSigDB (https://www.gsea-msigdb.org/gsea/msigdb/index.jsp) to generate the list of immunorelevant genes (28). To obtain the final list, all genes from the 4872 gene sets were merged and duplicates were removed (**Supplementary Table S1**). In order to identify potential immunoregulatory E2F1 target genes we subsequently aligned both lists with the DEGs from the transcriptomics data. To explore the interaction between genes and their functional network, DEGs were uploaded to the STRING database (RRID: SCR_005223; https://string-db.org/) and the comprehensive score > 0.4 was used as the cut-off standard to construct the networks which were uploaded to Cytoscape (3.10.2) to perform Gene Ontology (GO) and KEGG (RRID: SCR_012773) pathway enrichment analysis of the network members. Nodes represent genes and edges represent interactions. To obtain GO and pathways enrichment analyses from DEGs of the coculture experiments, we selected genes with a |Δ|>1.5-fold cut-off and uploaded them to the Database for Annotation, Visualization, and Integrated Discovery (DAVID; https://david.ncifcrf.gov/) (29, 30). Results with P < 0.05 were considered statistically significant.

### 2.16 Tumor immune infiltration analysis

The Tumor Immune Estimation Resource database (TIMER 2.0; http://timer.cistrome.org/) offers a web interface to study the molecular characteristics of tumor immune interaction (31). TIMER can assess the abundance of several infiltrating immune cell types based on gene expression profiles from TCGA. To investigate the immune infiltration of genes in melanoma (SKCM), TIMER and the integrated XCELL deconvolution was used to estimate the levels specifically for Th1, Th2, monocytes, eosinophils, and neutrophils in correlation with the expression of selected genes in primary and metastatic melanoma patient samples. P< 0.05 was considered to be statistically significant.

### 2.17 Mathematical modeling

We utilized previously published models (32, 33) to derive a qualitative mathematical model in ordinary differential equations describing the activation of the signaling pathway IL-6/IL6R/STAT3-E2F1. Information from the literature, normalization, and model assumptions were used to assign values to the model parameters. We created two instances of the model, one reflecting the activation of the pathway in melanoma cells, and another for the activation in CD4^+^ T cells, which differ in the expression levels attributed to the components of the IL-6 signaling pathway as indicated by the transcriptomic data from monoculture experiments. For the case of simulating melanoma cells, we set up the model to a switch-off configuration, modified iteratively the values of E2F1 expression in the model (from 0.1 to 100 a.u.), performed simulations, and computed the values of IL-6 at time 100 h. The procedure was repeated but modifying iteratively the expression levels of E2F1 and STAT3 (from 0.1 to 10 a.u.), and computed the values of IL-6 at time 100 h. To simulate CD4^+^ T cells, we configured the model to a switch-off state. The model was implemented in Octave (RRID: SCR_014398) and is available on Zenodo (RRID: SCR_004129; https://zenodo.org/doi/10.5281/zenodo.10777251).

### 2.18 Statistical analysis

Statistical significance was calculated by paired Student’s t-test (two-sided). All experiments were performed at least in triplicate. If not stated otherwise data are presented as mean ± standard deviations (SD). P values less than 0.05 were considered as significant.

## 3 Results

### 3.1 Identification of E2F1-induced immunoregulatory network in metastatic melanoma

High levels of E2F1 have been found in invasive and metastatic tumors, associated with unfavorable patient outcomes (14, 33). Abundant expression of the TF leads to a functional switch by rewiring GRNs that promote resistance to cancer therapies (18, 33), angiogenesis, extravasation, and EMT (15, 17). In addition, E2F1 contributes to the manifestation of cancer stem cells, linking E2F-dependent processes with immune cell crosstalk in cancer progression (34). However, little is known about the impact of E2F1 on immunoregulatory genes and its effects on TIME.

**Figure 1:**
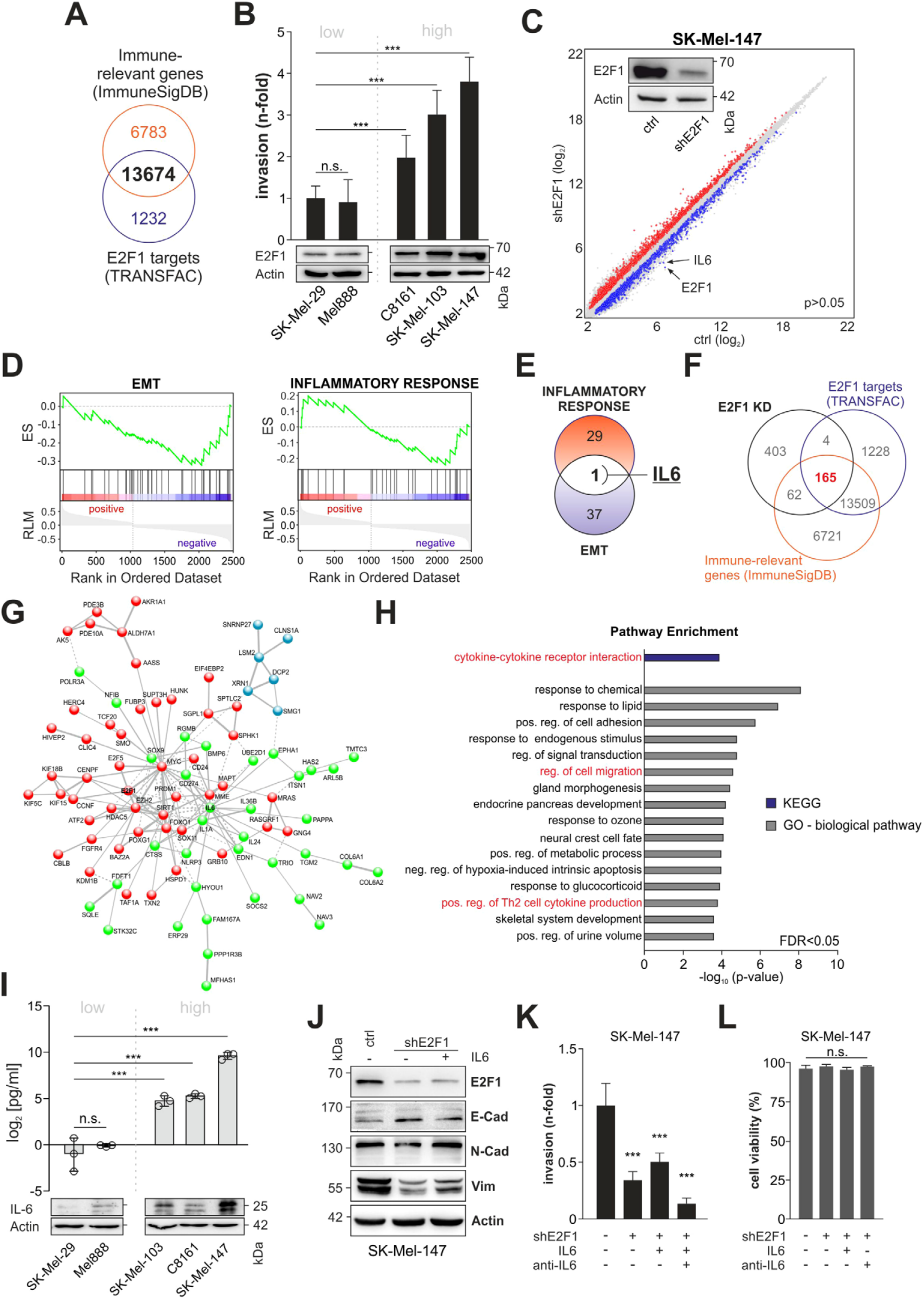
Identification of the E2F1-induced immunoregulatory network in metastatic melanoma. **(A)** Comparison of the genomic DNA-binding profile of E2F1 (TRANSFAC) with a gene list related to immunregulatory functions (ImmuneSigDB). **(B)** E2F1 expression levels correlate with the invasiveness in several melanoma cell lines determined by Matrigel assay. **(C)** Transcriptome of control (ctrl) and shE2F1 cell lines were compared to identify DEGs using cut-off values for P<0.05. Western blot confirmed knockdown of E2F1. Actin was used as loading control. **(D)** GSEA of genes differentially expressed in SK-Mel147 E2F1-KD mRNA microarrays show that EMT (left) and Inflammatory response are suppressed. **(E)** Venn diagram depicting the comparison of DEGs enriched in EMT and inflammatory response. **(F)** Downregulated genes (FC<-1.5, P<0.05) were filtered against the lists from **(A)** identifying 165 E2F1 regulated target genes with potential immunregulatory function. **(G)** STRING analyses show a highly interactive network composed of 89 of the 165 genes. K-means clustering reveals three interconnected sub clusters (red, blue, green). Within the green cluster IL6 has a hub position. **(H)** Functional analysis of the IL6 subcluster using GO and KEGG pathway enrichment. **(I)** High IL-6 expression and secretion in highly invasive melanoma cell lines were validated with ELISA and Western blot. **(J)** EMT marker expression in control and stable E2F1-KD cells with or without addition of IL-6. **(K)** Trans-well migration assay after depletion of E2F1 in aggressive melanoma cells. E2F1-KD cells were treated in parallel with recombinant I-L6 or IL-6 inhibitor (anti-IL-6) and compared to the control. **(L)** Determination of cell viability after treatment with IL-6 or anti-IL-6. n.s., not significant, *** P < 0.001; ** P < 0.01; * P < 0.05.

To evaluate E2F1’s potential to activate relevant immune-related factors, we compiled a list of genes involved in immunomodulation and cytokine production/secretion and screened them for E2F1 BS. Out of 20,457 immune-related genes identified, 13,674 (66.8%) were found to harbor an E2F1 BS (**Figure 1A**). For gene expression analyses, we used a human melanoma tissue culture model comprising different cell lines with characteristic invasive/metastatic potential that show a positive correlation between E2F1 levels and cell motility (**Figure 1B**). After generating a stable E2F1 knockdown (E2F1-KD) in the aggressive cell lines transcriptome arrays were performed, which revealed 3687 significantly differentially expressed genes (DEGs) compared to parental controls (**Figure 1C**, P=0.05). Applying GSEA on the DEGs showed that the gene sets for EMT and inflammatory response are suppressed upon knockdown of E2F1 in melanoma (**Figure 1D**), implicating a pleiotropic effect of E2F1-regulated genes that combines its prometastatic and immunomodulatory activity. Notably, IL6 emerged as the only candidate after comparisons of enriched genes from both hallmark sets (**Figure 1E**).

To further elucidate the effect of the TF, we combined the lists from Transfac and the immune-relevant genes (**Figure 1A**) with our transcriptomic data and identified 165 significantly downregulated genes that bear a putative impact on immune regulation (**Figure 1F**). STRING analysis of the identified genes demonstrated a highly interconnected network including E2F1, MYC, CD274 (PD-L1), EZH2, and IL6 (**Figure 1G**). Network clustering revealed three interconnected subgroups, two of which are related to biological processes such as RNA metabolism (blue subcluster), gene expression (green subcluster) and signal transduction (red subcluster, **Supplementary Table S2**). Interestingly, IL6, which exhibits the strongest reduction after E2F1 ablation (**Figure 1C**, -3.3 and -4.5-fold, respectively) occupies a central hub position in the gene expression subcluster (**Figure 1G**, green) associated with cytokine-mediated signaling, regulation of Th2 cytokine production and cell migration (**Figure 1H**, **Supplementary Table S2**). These data support a functional link between E2F1 and immune regulation, and strongly suggest that IL-6 is a key factor of the immunomodulatory activity exerted by this transcription factor.

Subsequent analysis of IL-6 expression and secretion clearly verified the positive correlation with endogenous E2F1 expression (**Figure 1I**). As production of IL-6 can enhance invasion and EMT (35), and given the observed pathway enrichment for ‘regulation of cell migration’ in the IL6 cluster (**Figure 1H**), we speculated that this cytokine could be involved in E2F1’s metastatic activity. Therefore, we examined the expression of both, E2F1 and IL-6, and mesenchymal or epithelial cell markers in aggressive melanoma cells, and analyzed their correlation (**Figure 1J**). Importantly, there was a positive correlation between E2F1/IL-6 (high, control) and N-cadherin/vimentin expression and a negative correlation with E-cadherin. After loss of E2F1, IL-6, N-cadherin, and vimentin decreased while E-cadherin increased. In turn, IL-6 treatment of E2F1-depleted cell lines could restore the N-cadherin and vimentin levels but reduced E-cadherin expression (**Figure 1J**). Accordingly, matrigel assays demonstrated that IL-6 treatment of shE2F1-expressing melanoma cells rescues their invasive capability, whereas addition of the IL-6 inhibitor led to the strongest reduction of cell motility (**Figure 1K**). Neither IL-6 nor the IL-6 inhibitor affected the cell lines’ viability (**Figure 1L**). These data strongly suggest that IL-6 contributes to E2F1-induced metastasis by enhancing EMT and invasiveness of melanoma cells.

### 3.2 IL-6 is a direct transcriptional target of the E2F1-STAT3 activator complex

**Figure 2:**
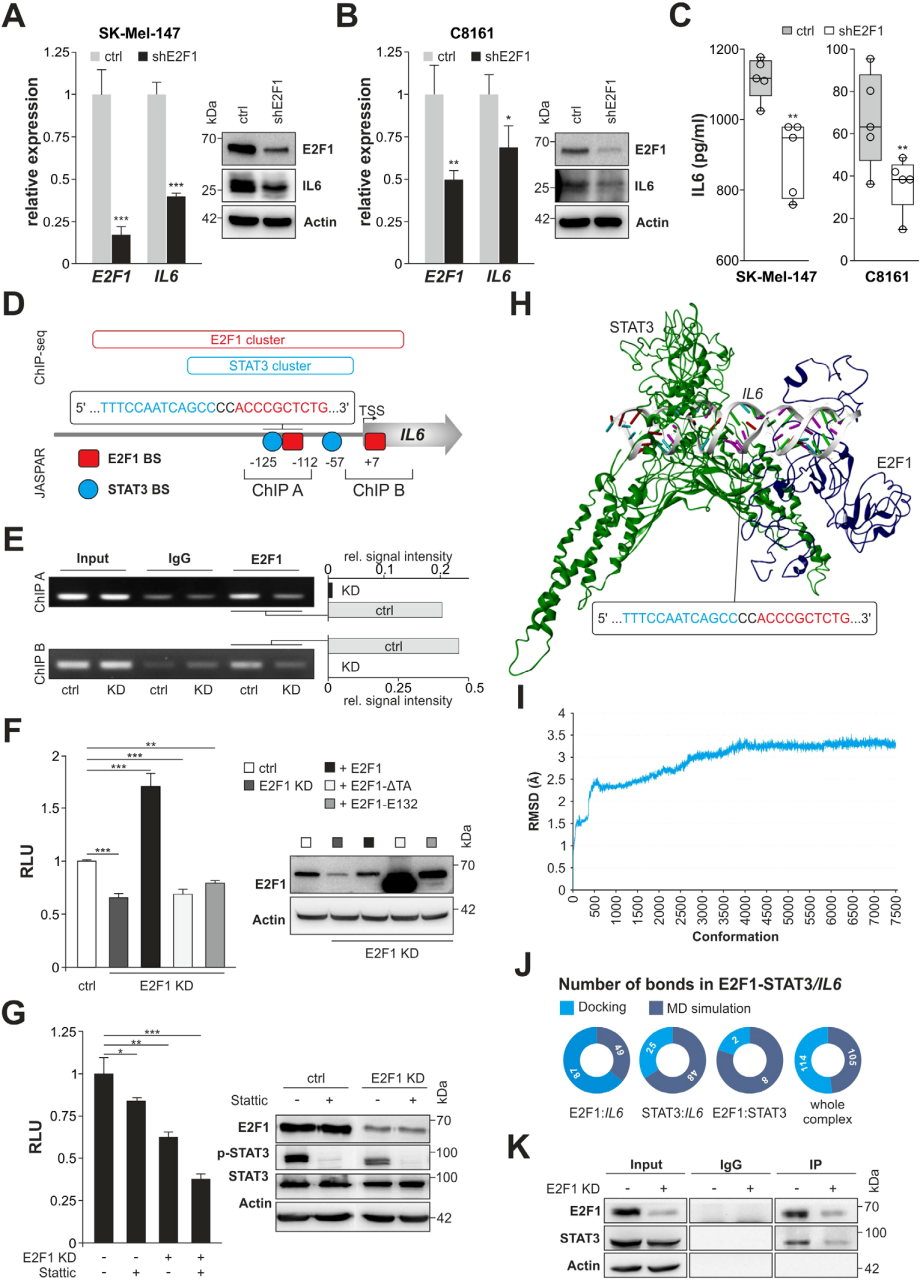
E2F1 interacts with STAT3 on the IL6 promoter regulating its expression. **(A, B)** Knockdown of E2F1 diminishes IL-6 expression on RNA and protein level in SK-Mel-147 **(A)** and C8161 **(B)**. **(C)** Reduced secretion of IL-6 in shE2F1 expressing melanoma cell lines (n=5). **(D)** Schematic diagram of the IL6 promoter including E2F1 and STAT3 ChIP seq clusters (https://genome.ucsc.edu) and the newly identified binding sites (BS) predicted by JASPAR (https://jaspar.elixir.no/). Additionally, ChIP fragments used for analysis is depicted with their corresponding sizes. Indicated positions are relative to the transcription start site (TSS). **(E)** ChIP assay was performed using IgG as negative control; input represents 10% of sheared chromatin. Bar graphs show quantification signal intensity of E2F1 bands normalized to input. **(F, G)** Luciferase activity of the IL6 promoter after transfection with E2F1 and mutants E132 or ΔTA in SK-Mel147 E2F1-KD **(F)** or in the presence or absence of E2F1 and inhibition of STAT3 (Stattic) **(G)**. Untreated cell lines were set as 1. Immunoblots confirmed E2F1 and STAT3 expression. **(H)** Representation of the final 3D model of E2F1-STAT3/IL6 promoter complex. **(I)** Illustration of the root mean square deviation (RMSD) from the docked complex throughout the molecular dynamics simulation. **(J)** Pie charts of changes in bond formation inside the E2F1-STAT3-IL6 promoter complex before and after molecular dynamics simulation. **(K)** SK-Mel-147 cell lysates expressing shctrl or shE2F1 were immunoprecipitated (IP) with anti-E2F1 antibody and immunocomplexes were blotted with anti-E2F1 and anti-STAT3. Actin was used as loading control; input represents 10% of total cell lysate. n.s., not significant, *** P < 0.001; ** P < 0.01; * P < 0.05.

To investigate the mechanism underlying a connection between E2F1 and IL-6, we examined both factors in high-E2F1/high-invasive SK-Mel-147 and C8161 vs. their E2F1-KD counterparts. Depletion of E2F1 significantly reduced IL6 transcripts in both cell lines as confirmed by Western blot and ELISA demonstrating decreased expression and secretion of IL-6 (**Figures 2A–C**). To further assess transcriptional regulation of IL6 we searched for E2F1 binding clusters using ChIP-seq data (https://genome.ucsc.edu/) by screening a 10,000 bp stretch upstream of the IL6 gene. We found a major E2F1 cluster at the center of a transcriptionally active region proximal to the translational start site. Notably, this region also contains a STAT3 cluster (**Figure 2D**), which harbors earlier described STAT3 BS (36). As it has been reported that STAT3 and E2F1 can generate transcriptional synergy depending on the cell context (37, 38), we applied JASPAR to the promoter sequence and identified a segment within the STAT3 cluster where the E2F1 binding motif is located in close proximity to a STAT3 BS, situated 112-125 base pairs upstream of the transcription start site (**Figure 2D**). The specific binding of E2F1 to this BS was confirmed by ChIP (**Figure 2E**, ChIP A), using another E2F1 BS at position +7 of the E2F1 cluster as control (ChIP B). For both newly detected E2F1 motifs, a strongly reduced binding was visible after E2F1 knockdown (**Figure 2E**). In addition, luciferase reporter assays revealed a significant decrease of IL6 promoter activity in E2F1-depleted vs. the parental cells (**Figure 2F**, left). Conversely, cotransfection of E2F1 into E2F1-KD cells induced a strong promoter reactivation, which was not observed after overexpression of DNA binding- (E132) or transactivation-deficient (TA) E2F1 mutants (**Figure 2F**). Strikingly, regarding a potential interaction between E2F1 and STAT3 due to spatial proximity of both BS (**Figure 2D**, ChIP A), we found that inhibition of each transcription factor alone only leads to partial reduction of promoter activity, whereas concomitant E2F1-KD together with STAT3 blockade causes almost complete loss of the luciferase signal (**Figure 2G**). These results ultimately emphasize a cooperative synergistic effect between E2F1 and STAT3 on the IL6 promoter.

Further, we examined whether both TFs establish a PPI on the target promoter. To this end, using above sequence data we designed a 3D model of the IL6 promoter region that comprises the neighboring BS (**Figure 2D**) and performed molecular docking studies with E2F1 and STAT3. The interaction of both TFs leads to the formation of a particularly stable E2F1-STAT3/IL6 promoter complex (**Figure 2H**), which has a high number of total interactions (**Supplementary Table S3**) and, compared to STAT3 alone, a significantly enhanced docking score of -237.31 kcal/mol (STAT3 dock score: -133.03 kcal/mol). Furthermore, the study revealed that the E2F1 region encompassing amino acids 110-194, is responsible for protein-DNA and protein-protein interactions (**Supplementary Table S3**). To then assess the flexibility and stability of the E2F1-STAT3/IL6 promoter complex, we simulated time-dependent molecular dynamics (MD) using DS 2022. The analyses incorporated the Root Mean Square Deviation and showed an initial deviation of 3.2 Å in the first eight ns (up to 4,000 conformations), which subsequently converged without further deviations (**Figure 2I**). We also extracted binding information of the complex from the last frames of the simulation and compared them with the initial condition. In addition, we investigated alternative scenarios with only two factors, namely E2F1:IL6, STAT3:IL6, and E2F1:STAT3. In summary, bond formation increased in STAT3:IL6 and E2F1:STAT3 complexes (25 vs. 48 and 2 vs. 8, respectively), which contributed to the stability of the docked complex. For E2F1:IL6 and the entire complex, the number of bonds decreased during the MD simulation (87 vs. 49 and 114 vs. 105) (**Figure 2J**, **Supplementary Table S4**). However, the overall stability of the E2F1-STAT3/IL6 promoter complex is mainly attributed to the bond formation between STAT3 and E2F1. The results of the *in silico* docking analyses were confirmed by IP experiments. As demonstrated in **Figure 2K**, STAT3 was clearly recovered from E2F1 immunoprecipitates of whole melanoma cell lysates, whereas knockdown of E2F1 resulted in decreased binding of STAT3.

### 3.3 E2F1-dependent melanoma-T cell crosstalk modulates transcriptional landscape boosting IL-6 expression and disrupting anticancer immunity pathways

**Figure 3:**
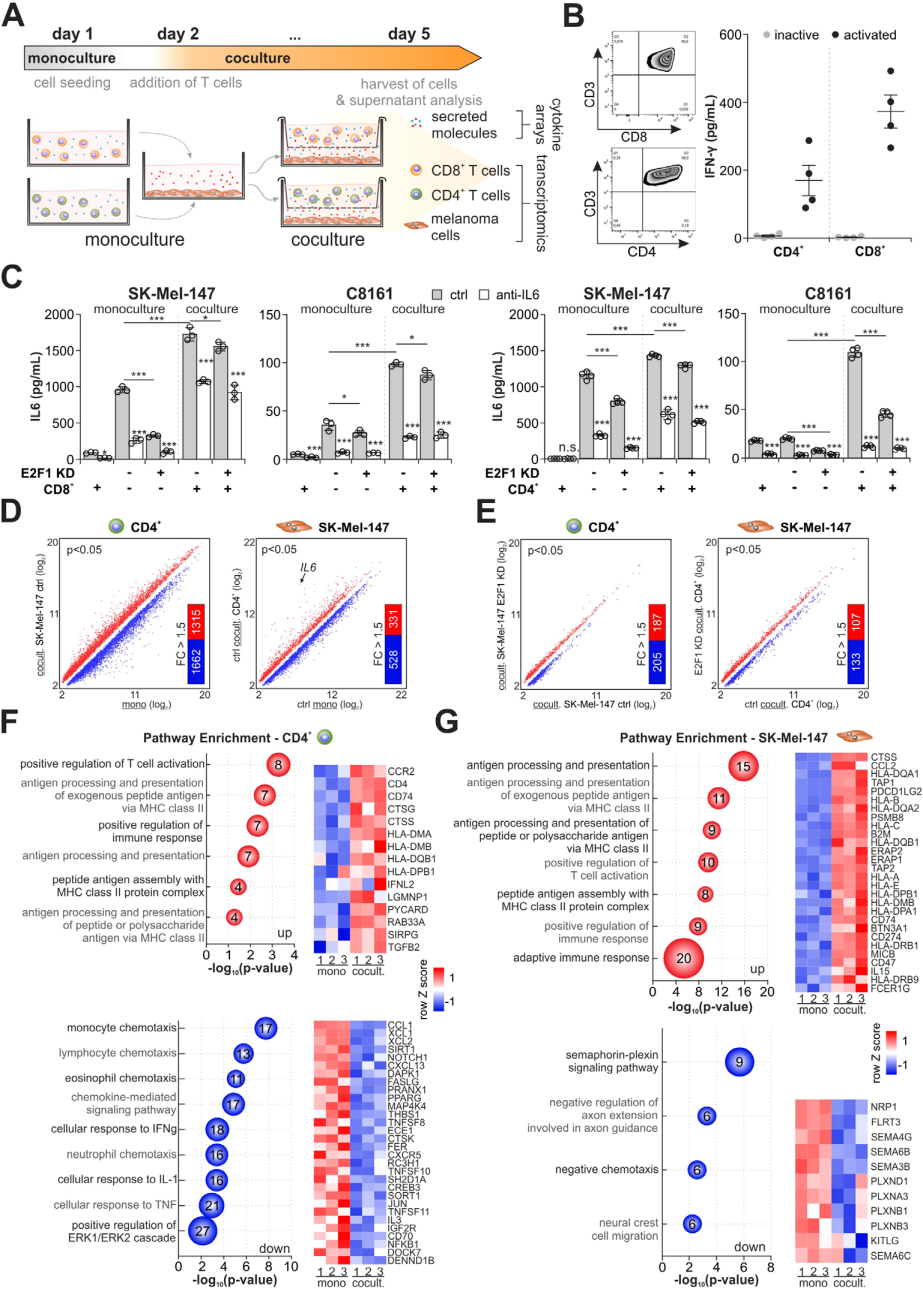
Influence of the E2F1-dependent crosstalk on the transcriptional landscape in melanoma and CD4^+^ T cells. **(A)** Workflow and culture conditions of highly aggressive melanoma with T cells. Separated by a membrane, CD4^+^ or CD8^+^ T cells were indirectly cocultured with SK-Mel-147 or C8161 expressing shctrl or shE2F1. Monocultured cells served as reference to their cocultured counterparts. **(B)** Left: Representative flow cytometry images of isolated T cell populations. Right: IFN-γ levels were determined by ELISA to verify activation of CD8^+^ and CD4^+^ T cells from four different donors used in the coculture experiments. **(C)** IL-6 secretion was quantified in mono- and cocultured immune and control or E2F1-KD cancer cells in the presence or absence of an anti-IL-6. **(D, E)** Expression analysis in CD4^+^ cells cocultured with high-E2F1 melanoma cells (ctrl) compared to CD4^+^ monoculture (**D**, left) and SK-Mel-147 cocultured with CD4^+^ cells compared to SK-Mel-147 monoculture (**D**, right) and **(E)** transcriptomes of cocultured CD4^+^ (left) and SK-Mel-147 E2F1-KD cells (right) in comparison to their respective coculture condition with high-E2F1 (ctrl). Scatter plots show significantly differentially expressed genes (P<0.05) while bar graphs depict the amount of annotated genes with a 1.5-fold change (FC) subjected to GO analysis. **(F, G)** Bubble blots demonstrate the most enriched groups and their GO terms for biological processes of up- and downregulated genes of cocultured CD4^+^ **(F)** and SK-Mel-147 ctrl **(G)** lines ranked by most significant P-values (-log_10_, x-axis). Bubble size and numbers indicate the amount of related genes. *** P < 0.001; ** P < 0.01; * P < 0.05.

Since E2F1 orchestrates the immunomodulatory network via IL-6 (**Figure 1G**), we first investigated the effect of IL-6 on the cancer-immune crosstalk. We established an indirect coculture system that permits cytokine communication while preventing direct contact between cancer and immune cells (39). As shown in **Figure 3A**, we used high-E2F1 vs. E2F1-KD melanoma cell lines grown as monocultures or together with either CD4^+^ or CD8^+^ T cells from healthy donors. To support T cell activation, a minimal doses of 0.25 ug/ml PHA (40) and 20 U/ml IL2 were added to the medium and their activity was validated by measuring PD1 expression (41) and IFN-γ secretion (42). Viability of cocultured melanoma and immune cells was verified by live/dead staining (**Figure 3B**, **Supplementary Figures S1A, B**).

After three days of culturing, the amounts of IL-6 secreted into the supernatants were quantified. In contrast to monocultured CD8^+^ (left panels) or CD4^+^ (right panels) T cells, metastatic melanoma cells showed a high IL-6 concentration in the supernatant (**Figure 3C**). Importantly, IL-6 levels considerably increased when cancer and immune cells were cultured together, suggesting a positive feedback loop between melanoma and T cells (43). Although depletion of E2F1 mainly counteracts this effect, IL-6 is still detectable under coculture conditions and could only be reduced by direct inhibition. In fact, combined treatment with IL-6 inhibitor and E2F1-KD was necessary to diminish the elevated cytokine levels in the presence of T cells (**Figure 3C**) without affecting cell viability (**Supplementary Figures S1C, D**).

Due to the impact of E2F1 on GRNs in cancer cells and the observed intriguing changes in IL-6 secretion, we wondered to what extent gene expression profiles of melanoma and T cells are altered, and whether E2F1-KD can positively influence the genomic program of T cells in the sense of an antitumor immune response. Thus, we performed high-throughput array analyses of cancer and immune cells from mono- and coculture experiments (**Figure 3A**).

Comparison of transcriptomes from cocultured CD4^+^ T cells and SK-Mel-147 ctrl vs. their corresponding monocultures revealed a total of 9,453 and 4,521 DEGs (P<0.05, **Figure 3D**), indicating a strong reciprocal induction of transcriptional changes in E2F1-abundant melanoma and immune cells through a cytokine-mediated crosstalk. It is notable that IL6 is most strongly expressed in melanoma cells under coculture conditions (**Figure 3D**, right), which also verifies the detected increase in IL-6 secretion shown in **Figure 3C** (right panels) and supports the presence of an active feedback loop. E2F1 knockdown severely reduced these alterations in gene expression in both cell types to 1,462 (CD4^+^) and 1,708 (SK-Mel-47 E2F1-KD) DEGs, respectively (**Figure 3E**), underlining the regulatory role of E2F1 in the crosstalk between tumor and immune cells.

To further investigate the potential biological functions of the identified genes, we selected fully annotated DEGs with a |Δ|>1.5-fold cut-off (**Figure 3D**) and performed GO enrichment analyses. We found that 1,315 upregulated genes in CD4^+^ cocultured with SK-Mel-147 are primarily associated with processes and pathways like T cell activation and increased MHC-II-related responses (i.e. antigen processing and presentation) (**Figure 3F**, top), indicating T cell functionality (44). In contrast, 1,662 downregulated genes are related to chemotaxis of monocytes, eosinophils, and neutrophils (**Figure 3F**, bottom), suggesting an impaired directed communication with innate immune cells. Importantly, the analysis also revealed repression of genes associated with responses to IFN-γ and TNF-α, two pivotal mediators of CD4^+^ Th1 differentiation that are known to promote an antitumor response (45). This is in line with the repression of genes related to ERK1/ERK2 pathways (**Figure 3F**, bottom) that have been connected with Th1 development (46). In melanoma cells, we found high scores for antigen processing/presentation and positive regulation of the immune response from 331 upregulated genes (**Figure 3G**, top) which is consistent with the highly active immune phenotype of melanoma (47), whereas downregulated genes are mainly involved in neuronal processes (**Figure 3G**, bottom). Surprisingly, among the upregulated immune activating genes, we identified several immunosuppressive factors like CCL2, a potent chemoattractant for Th2 cells, and checkpoint inhibitor CD274 (**Figure 3G**, top), which is, as shown in **Figure 1G** connected to the identified immunomodulatory E2F1 network via the IL6 cluster.

### 3.4 Tumor infiltration of CD4^+^ Th2 cells in melanoma patients and immunosuppressive type-2 cytokine secretome correlates with an activated E2F1/IL-6 axis

**Figure 4:**
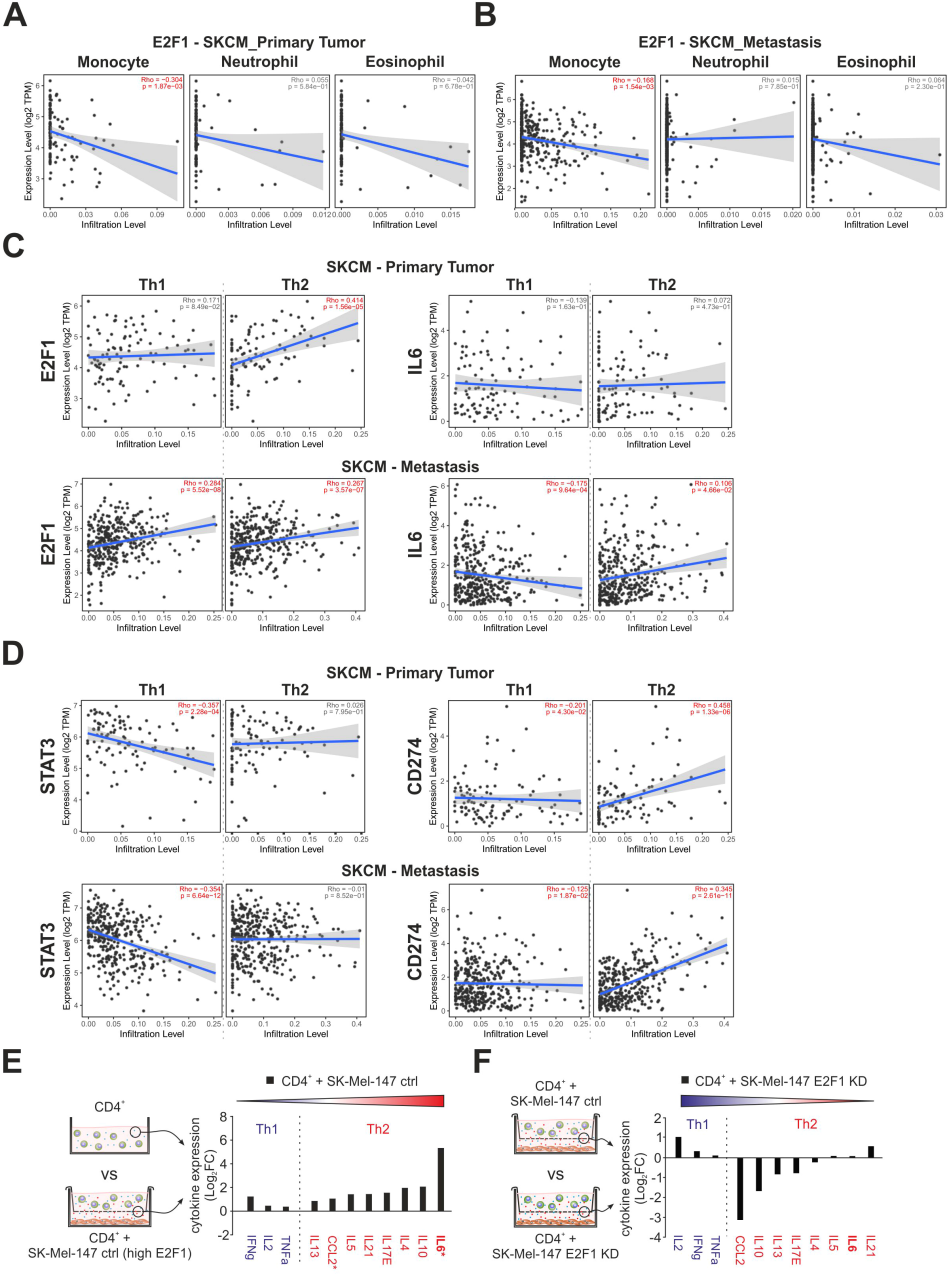
The impact of E2F1 on immune cell infiltration and cytokine release in coculture with T cells. **(A, B)** Correlation of E2F1 expression with infiltration of monocytes, eosinophils, and neutrophils in melanoma patients with primary tumors **(A)** or metastasis **(B)**. **(C, D)** Estimation of CD4^+^ Th1 and Th2 cell infiltration cells in melanoma patients with primary tumors or metastasis correlated to the expression of E2F1, IL-6 **(C)**, and STAT3, CD274 **(D)**. Significant correlations are highlighted in red. **(E, F)** Cytokine assays of supernatants from different culture/coculture conditions were compared as illustrated showing an E2F1-dependent increased secretion of immunosuppressive type 2 cytokines **(E)** that was completely reversed after E2F1-KD in melanoma cells **(F)**. Asterisks indicate transcriptionally upregulated factors. FC, fold change.

The TIME is a complex and dynamic ecosystem in which diverse populations of tumor and immune cells coexist. Considering the impact of E2F1 on the surroundings via the tremendous upregulation of IL-6 and the ambivalent alteration of the transcriptional landscape, we analyzed the potential correlation between the expression of E2F1 and tumor infiltration of immune cells in primary and metastatic skin cancer using TIMER 2.0. Based on functional annotations and pathway enrichment (**Figure 3F**), we first investigated the abundancies of monocytes, neutrophils, and eosinophils. Interestingly, we found that E2F1 had a limited negative correlation with monocytes in both primary (**Figure 4A**, left) and metastatic (**Figure 4B**, left) patient samples. However, no significant presence of neutrophils or eosinophils was evident in both cancer groups (**Figures 4A, B**) corroborating our previous findings with downregulation of genes related to chemotaxis for these immune cell types (**Figure 3E**, bottom).

More importantly, our data evidently supported a strong influence of E2F1 and its target IL-6 on the Th1/Th2 development like Th2 cytokine production (**Figure 1H**), downregulation of ERK1/2 pathways, IFN-γ response (**Figure 3F**, bottom), or CCL2 expression in melanoma cells (**Figure 3G**). Surprisingly, E2F1 showed a significant positive correlation for Th2 infiltration in the primary tumor, while IL6 had no effect (**Figure 4C**, top). In contrast metastatic samples exhibit only a moderate correlation with Th2 cells, but also with Th1, which could support the observation of the cooccurrence of immunostimulatory and immunosuppressive mechanisms (47). However, Th2 prevalence could be further promoted by IL6, as indicated by an increased abundance of Th2 and a concomitant decrease of Th1 cells in advanced melanoma (**Figure 4C**, bottom). This shift is further amplified via the expression of E2F1’s cofactor STAT3, which consistently suppresses Th1. Furthermore, CD274 simultaneously blocks Th1 and promotes Th2 infiltration in both, primary and metastatic tumors (**Figure 4D**).

To validate the E2F1-dependent Th2 shift, cytokine arrays were performed with supernatants obtained from melanoma-immune cell cocultures, with a particular focus on type-1 and type-2 cytokines (**Figures 4E, F**). As illustrated in **Figure 4E**, the supernatant of CD4^+^-SK-Mel-147 coculture revealed a profound increase for all detected type-2 cytokines, with IL-6 showing the strongest response. Strikingly, loss of E2F1 resulted in a remarkable reduction of most pro-inflammatory proteins, but without affecting the type-1 cytokines (**Figure 4F**). Consistent with our previous data, the dramatically elevated IL-6 levels measured in high-E2F1 melanoma cocultures (**Figure 4E**) substantially decreased under E2F1-KD (**Figure 4F**). These results provide strong evidence that increased E2F1/IL-6 expression triggers Th2-driven inflammation via type-2 cytokines, whereas Th1 cell activity is suppressed, establishing an immunosuppressive environment.

### 3.5 Mathematical model predicts E2F1-dependent mechanisms of cancer-immune crosstalk

**Figure 5:**
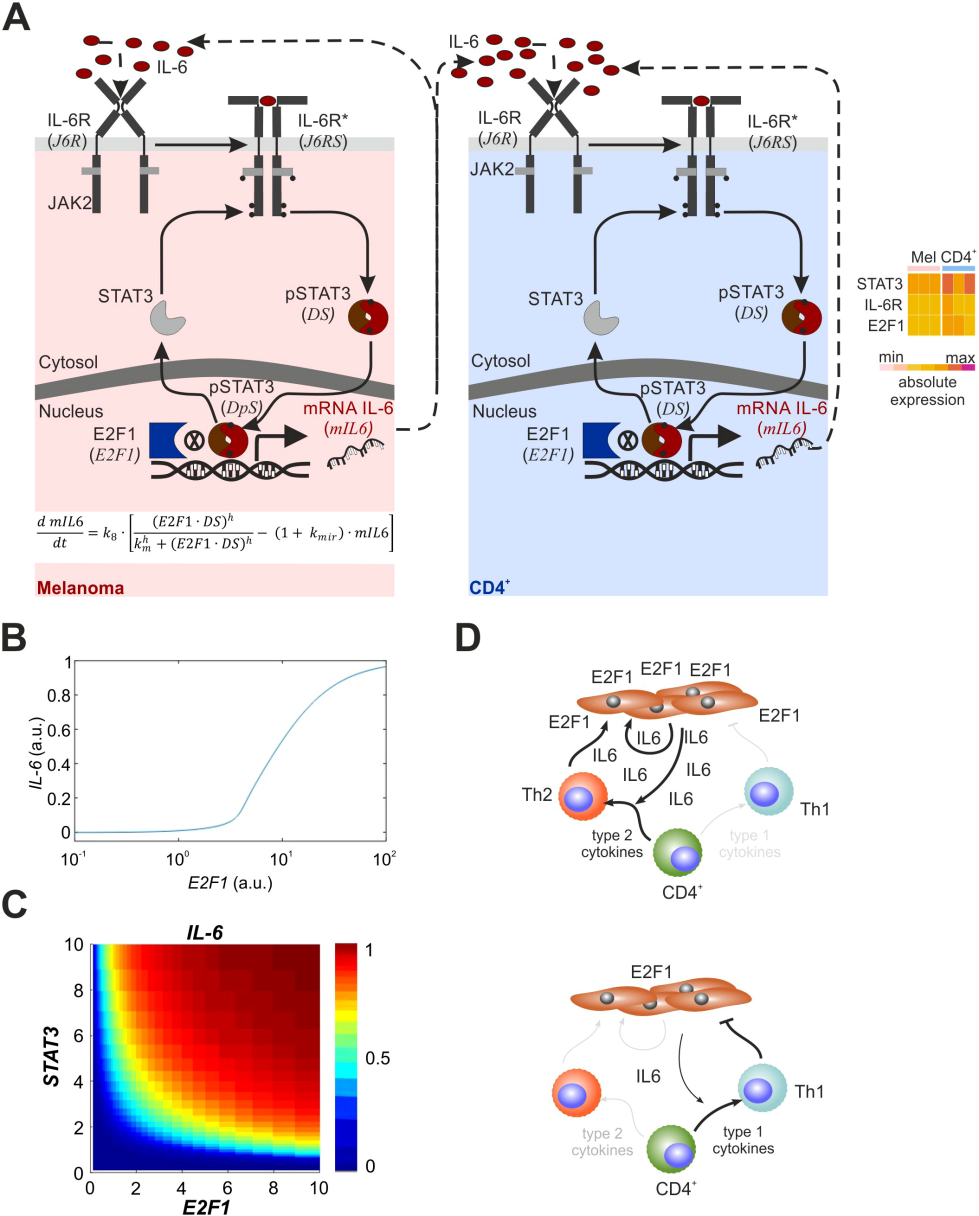
Mathematical model and overview of the melanoma-CD4^+^ T cell crosstalk with E2F1-STAT3/IL-6/IL6R axis. **(A)** Mathematical model of the E2F1-STAT3/IL-6 axis including the auto- and paracrine feedback loops from melanoma and immune cells. The heatmap shows that factors related to the IL6R-STAT3-E2F1 pathway are expressed in melanoma and CD4^+^ T cells prior to activation and crosstalk. **(B)** Crosstalk simulation illustrating IL-6 expression in melanoma and CD4^+^ T cells. **(C)** Output of simulation representing expression levels of E2F1 or STAT3 that are sufficient to trigger IL-6 secretion in melanoma cells. **(D)** The impact from E2F1 on the melanoma T cell crosstalk where high E2F1 expression leads to an immunosuppressive TME through feedback loops triggered increased release of IL-6 inducing a Th2 shift (left). In contrast, E2F1 depletion in melanoma cells blocks the augmented IL-6 secretion and, in turn, diminishes type-2 cytokine production favoring an anti-tumor Th1 response.

According to **Figures 4C, D**, STAT3, which cooperates with E2F1 in IL-6 expression, critically contributes to the Th2/Th1 balance in melanoma patients. To verify the dynamics of the molecular mechanism and the cancer immune crosstalk, we finally derived a mathematical ordinary differential equation (ODE) model based on the core cancer-immune interaction network that describes the activation of the E2F1-STAT3/IL-6/IL6R signaling pathway and consequences of the interplay between E2F1 and STAT3 on transcriptional regulation of IL-6 (auto- and paracrine activation; **Figure 5A**). We created different versions of the model to represent the regulation of the pathway in melanoma and CD4^+^ T cells. Based on our analysis of TF binding sites and current literature (37, 38), we assumed that IL6 transcription is controlled synergistically by E2F1 and active STAT3. Also, the coculture system indicates that IL-6 secreted by melanoma cells to the tumor niche activates in a paracrine manner CD4^+^ cells in their vicinity. We performed iterative simulations modifying the values of E2F1 expression and found, that under basal STAT3 activation, increasing levels of E2F1 in melanoma cells can trigger the expression and secretion of IL-6 (**Figure 5B**). Our simulations suggest that high levels of IL-6 secretion by melanoma cells can be either achieved through high levels of E2F1, high levels of STAT3, or moderate levels of both TFs (**Figure 5C**).

In addition, we simulated the effect of IL-6 secreted by melanoma on CD4^+^ T cells present in the TME (**Figure 5A**, right). The simulations propose that IL-6 secreted by high-E2F1 melanoma cells is sufficient to trigger the activation of the IL-6/IL6R/STAT3 axis in CD4^+^ immune cells, which could also trigger secretion of other inflammatory factors. Importantly, this model displayed ultrasensitivity in the CD4^+^ response induced by IL-6-mediated autocrine activation and thus, enables a therapy-oriented interpretation of the patient data.

## 4 Discussion

Patients with malignant melanoma and other advanced cancers have in recent years significantly benefited from ICI treatments. Nevertheless, some patients are refractory or resistant to these therapies most likely due to immune inhibitory soluble and cellular components within the TME that promote tumor progression and facilitate the immune escape of cancer cells (48). As such, melanoma is one of the most heterogeneous cancers in terms of histology, genomic alterations, gene expression patterns, and metastatic behavior. The search for triggers and clinically useful markers of metastasis revealed that E2F1 critically orchestrates epigenetic cancer evolution and therapy resistance (14, 15, 33). Assuming that changes of the immune niche by tumor-intrinsically elevated E2F1 expression could be mechanistically crucial for its prometastatic function, which also impairs the virtue of immunotherapies, the influence of this TF on the communication between highly aggressive melanoma and healthy donor-derived T cells was comprehensively investigated by an integrative approach combining cell coculture models, high-throughput analyses, and structure-based modeling.

We report here that E2F1 physically interacts with active STAT3 and synergistically controls IL-6 expression to induce a tumor-associated inflammatory GRN that ultimately mediates metastatic traits and modulates the TME by releasing immunomodulatory factors. Data-based simulations revealed that high levels of E2F1 in melanoma cells trigger autoactivation and a paracrine positive feedback loop with CD4^+^ T cells via secretion of IL-6 to the tumor niche to generate an inflammatory secretome. This leads to the exacerbation of intercellular cytokine signaling including IL-6 and deregulation of the transcriptional landscape in immune and cancer cells. Our studies clearly show that E2F1-STAT3/IL-6 produced by skin cancer cells shifted the Th1/Th2 balance toward the Th2 phenotype, whereas depletion of E2F1 in turn caused a pronounced decrease in type-2 cytokines that potentially determines the development and responsiveness of Th1 cells (**Figure 5D**). Consistently, the clinical data revealed that melanoma with abundant E2F1, STAT3 and IL-6 preferentially induce and maintain infiltration of Th2, while simultaneously blocking Th1 cells. Thus, our experimental setting provides a mechanistic model to derive the immune response in melanoma patients and emphasizes the prognostic and therapeutic benefit of disrupting the E2F1 and/or STAT3 coregulator interaction and its networks.

An active immunological microenvironment with a high density of activated T cells has been associated with a favorable prognosis and a good response to immunotherapy (49). At the same time, the efficacy of the antitumor response or immune checkpoint inhibition hinges on maintaining a delicate balance between immunostimulatory and immunosuppressive mechanisms in the TME (47). In particular, this can be mediated by genomic dysregulation of oncogenic pathways (50, 51) and correlates with T cell exclusion, a process termed primary immune ignorance (47). According to our findings, E2F1, as a major driver of EMT signaling pathways and metastasis (14, 15) has the ability to control an immune GRN that includes well-characterized targets such as MYC, CD274, and IL6 to orchestrate the immunological landscape (52–54). Furthermore, our data point to a dynamic crosstalk between melanoma and CD4^+^ T cells leading to E2F1-dependent perturbations in the immunoregulatory transcriptome of both cell types. The upregulation of pro-inflammatory genes like IL6 is accompanied by the expression of immunosuppressive markers and inhibitory molecules, reflecting the simultaneous presence of opposing mechanisms that are mediated by E2F1.

Tipping the balance of immunosurveillance from tumor destruction to its promotion requires multiple signaling pathways that are impacted by the expression of cytokines from cancer cells, immune cells, and other cell types in the surrounding tissue (55). We demonstrate that high levels of E2F1 in melanoma cells disrupt the balance regulated by various cytokines/interleukins such as IL-6. This disruption negatively affects the diversity and differentiation of immune cells and leads to a TME that could be immunosuppressive and ultimately prometastatic. In particular, CD4^+^ T cells are crucial for cytokine-mediated control of the immune response. The plasticity and heterogeneity of these cells provide an equilibrium of pro- and anti-inflammatory mechanisms that ensures an appropriate immune response (56). In this respect, Th2, a specific subset of CD4^+^ T cells, have long been known to promote tumor growth and metastasis by suppressing the immune system (56).

Infiltration data from skin cancer patients confirmed the presence of Th2 cells with increased E2F1 expression in primary tumors, stressing the induction of a primary immune suppression (47). Regarding the influence of E2F1 on DNA repair and genetic instability (57), Tan and colleagues (2023) have recently shown that hyperactivation of E2F1-induced DNA replication stress in tumors promotes their mutational burden, which is associated with the infiltration of immunosuppressive Th2 and MDSCs (11). In patients with metastatic melanoma, however, E2F1 appears to exert a beneficial influence on Th1 infiltration, which may be associated with the activation of additional immunostimulatory mechanisms. Nevertheless, E2F1-induced expression of IL6 and CD274, or elevated levels of STAT3, can mitigate the effect nurturing toward the Th2 direction in advanced stages of melanoma. The dominance of the Th2 subset is also evident by the enriched levels of type-2 cytokines, including IL4 and IL10, in the coculture of cancer and immune cells. However, this effect on cytokine constellation was fully reversed when E2F1 is ablated in metastatic melanoma cells, while type-1 cytokines IL2, IFN-γ, and TNF-α maintained in the supernatant, potentially restoring a Th1 equilibrium.

In addition, CCL2, another critical factor in the TIME, was upregulated under coculture conditions and showed a strong response upon E2F1 knockdown. The chemokine is a potent chemoattractant for Th2 and Treg cells as well as a driver of metastasis and contributes to the differentiation of TAMs (58–61). This is in line with our previous findings that E2F1 promotes metastasis by enhancing the infiltration of TAMs via physical interaction of the TF with metastasis-associated protein MTA1 and induction of HAS2 target gene expression in pancreatic cells (19). Therefore, it is highly feasible that E2F1-KD negatively regulates chemoattractants in skin cancer, contributing to an antitumor immune response by blocking TAMs and Th2 cells.

A master player in the E2F1-induced cancer-immune cell crosstalk is IL-6. Deregulated, elevated expression of this pro-inflammatory cytokine is associated not only with chronic inflammation and autoimmune diseases, but also with tumor development, invasiveness and metastasis (62, 63). Beyond its pro-inflammatory properties, IL-6 is associated with anti-inflammatory functions that are depending on two signaling pathways. Classical IL-6 signaling stimulates target cells by binding to a membrane-bound complex of IL6R and glycoprotein 130 (gp130), which activates anti-inflammatory processes. In contrast, trans-signaling of IL-6 is mediated by binding to a soluble IL6R inducing pro-inflammatory responses (64). While gp130 is ubiquitously expressed, membrane-bound IL6R is mostly restricted to leukocytes and hepatocytes (65). This supports the potential to generate immunosuppression in an IL-6-enriched milieu, like macrophage differentiation, increased IgG production from activated B cells, or negative regulation of DCs maturation through STAT3 activation (66). Eventually, IL-6 promotes the polarization of CD4^+^ T cells into Th2 while inhibiting the Th1 phenotype (67), underscoring the immunosuppressive function of the E2F1-STAT3/IL-6 signaling axis.

Given IL-6’s pleiotropy as well as its cell- and dose-dependency, clinical application has proven difficult. Recent studies have shown that high IL-6 levels can impair the effectiveness of anti-PD-L1 and anti-CTLA-4 checkpoint inhibitors (68). Therefore, IL-6 blockade in combination with ICI can enhance the cytotoxic T lymphocyte response to the tumor and improve its control compared to ICI alone (69). IL-6 neutralization may allow patients to continue ICI treatment by counteracting cytokine release syndrome, an adverse effect that can occur from immune and chimeric antigen receptor (CAR)-T cell therapies (70). Subsequently, IL-6 levels can be used to predict the response to immune checkpoint therapy, and reducing IL-6 levels may enhance their efficacy (68). Of note, the combination of IL-6 neutralization with ICIs remains controversial, since inhibition of IL-6 has been reported to suppress PD-1 and PD-L1, thus attenuating the activity of ICIs (63). In fact, the balance in the TME involves more than one cytokine (71), which could be achieved by directly silencing tumor-associated E2F1.

The inclusion of E2F1, for instance, in the form of patient signatures, offers the opportunity to better assess the efficacy of cytokines like IL-6 or their inhibitors as therapeutic agents. High E2F1 levels in advanced melanoma favor a Th2 response. Lowering E2F1 expression or blocking the deleterious E2F1-STAT3/IL-6 axis to modulate the tumor secretome could therefore improve antitumor responses and reduce adverse effects when interleukins and ICIs are used in combination. Clinically, a significant proportion of patients with advanced melanoma develop autoimmunity, or existing autoimmune disease worsens on ICI (72). This suggests that E2F1 also plays a role as a predisposing factor for autoimmune comorbidities. Possible adverse consequences of cancer immunotherapy could be predicted based on the transcriptional signature and need to be clarified by further investigations.

A promising approach to disrupt the E2F1-STAT3/IL-6 axis could be the pertubation of the E2F1-STAT3 assembly. The ability of E2F1 to interact with certain transcriptional coregulators expands its functional portfolio, as previously demonstrated for metastasis-initiating and metabolic processes (18, 19). The physical association with STAT3 creates an immunosuppressive TME by promoting pro-metastatic properties such as invasiveness and EMT through the upregulation of IL-6. Since STAT3 itself is an important driver of tumor progression in various cancers and its constant phosphorylation leads to deregulated activity, therapeutic approaches have been pursued to block STAT3 function. Currently, the main strategy is to prevent the formation of functional dimers by interrupting phosphorylation (73, 74). However, none of these approaches have been clinically applied (74) The present study now offers new alternative solutions either by transcriptional perturbation or by disrupting the cooperation between E2F1 and the coregulator, in this case STAT3, which has the potential to rescue a cancer-refusing immune response by rebalancing the TME and removing prometastatic properties. Finally, computer models that describe the complexity and dynamics of cellular processes and predict disease progression equip scientists with powerful tools. By training these models with patient-derived omics data and high-resolution secretome profiles, design and accuracy of patient-specific drugs can be improved.

## Data Availability

The datasets presented in this study can be found in online repositories. The names of the repository/repositories and accession number(s) can be found below: https://www.ebi.ac.uk/arrayexpress/, E-MTAB-12750.

## References

[1] Blomberg OS Spagnuolo L de Visser KE. Immune regulation of metastasis: mechanistic insights and therapeutic opportunities. Dis Model Mech. (2018) 11. doi: 10.1242/dmm.036236 PMC6215427 30355585

[2] van Weverwijk A de Visser KE. Mechanisms driving the immunoregulatory function of cancer cells. Nat Rev Cancer. (2023) 23:193–215. doi: 10.1038/s41568-022-00544-4 36717668

[3] Kalaora S Nagler A Wargo JA Samuels Y. Mechanisms of immune activation and regulation: lessons from melanoma. Nat Rev Cancer. (2022) 22:195–207. doi: 10.1038/s41568-022-00442-9 35105962

[4] Passarelli A Mannavola F Stucci LS Tucci M Silvestris F. Immune system and melanoma biology: a balance between immunosurveillance and immune escape. Oncotarget. (2017) 8:106132–42. doi: 10.18632/oncotarget.22190 PMC5739707 29285320

[5] Verdegaal EM de Miranda NF Visser M Harryvan T van Buuren MM Andersen RS. Neoantigen landscape dynamics during human melanoma–T cell interactions. Nature. (2016) 536:91–5. doi: 10.1038/nature18945 27350335

[6] Carlino MS Larkin J Long GV. Immune checkpoint inhibitors in melanoma. Lancet. (2021) 398:1002–14. doi: 10.1016/S0140-6736(21)01206-X 34509219

[7] Fenton SE Sosman JA Chandra S. Resistance mechanisms in melanoma to immuneoncologic therapy with checkpoint inhibitors. Cancer Drug Resistance. (2019) 2:744. doi: 10.20517/cdr.2019.28 35582566 PMC8992532

[8] Simiczyjew A Dratkiewicz E Mazurkiewicz J Ziętek M Matkowski R Nowak D. The influence of tumor microenvironment on immune escape of melanoma. Int J Mol Sci. (2020) 21:8359. doi: 10.3390/ijms21218359 33171792 PMC7664679

[9] Sumimoto H Imabayashi F Iwata T Kawakami Y. The BRAF–MAPK signaling pathway is essential for cancer-immune evasion in human melanoma cells. J Exp Med. (2006) 203:1651–6. doi: 10.1084/jem.20051848 PMC2118331 16801397

[10] Luke JJ Bao R Sweis RF Spranger S Gajewski TF. WNT/β-catenin pathway activation correlates with immune exclusion across human cancers. Clin Cancer Res. (2019) 25:3074–83. doi: 10.1158/1078-0432.CCR-18-1942 PMC6522301 30635339

[11] Tan K Song Y Xu M You Z. Clinical evidence for a role of E2F1-induced replication stress in modulating tumor mutational burden and immune microenvironment. DNA Repair (Amst). (2023) 129:103531. doi: 10.1016/j.dnarep.2023.103531 37453246 PMC11847531

[12] Martin TD Patel RS Cook DR Choi MY Patil A Liang AC. The adaptive immune system is a major driver of selection for tumor suppressor gene inactivation. Science. (2021) 373:1327–35. doi: 10.1126/science.abg5784 PMC13397623 34529489

[13] Huang L Guo Y Liu S Wang H Zhu J Ou L. Targeting regulatory T cells for immunotherapy in melanoma. Mol BioMed. (2021) 2:11. doi: 10.1186/s43556-021-00038-z 34806028 PMC8591697

[14] Alla V Engelmann D Niemetz A Pahnke J Schmidt A Kunz M. E2F1 in melanoma progression and metastasis. J Natl Cancer Institute. (2010) 102:127–33. doi: 10.1093/jnci/djp458 20026813

[15] Khan FM Marquardt S Gupta SK Knoll S Schmitz U Spitschak A. Unraveling a tumor type-specific regulatory core underlying E2F1-mediated epithelial-mesenchymal transition to predict receptor protein signatures. Nat Commun. (2017) 8:198. doi: 10.1038/s41467-017-00268-2 28775339 PMC5543083

[16] Swiatnicki MR Andrechek ER. Metastasis is altered through multiple processes regulated by the E2F1 transcription factor. Sci Rep. (2021) 11:9502. doi: 10.1038/s41598-021-88924-y 33947907 PMC8097008

[17] Meier C Spitschak A Abshagen K Gupta S Mor JM Wolkenhauer O. Association of RHAMM with E2F1 promotes tumor cell extravasation by transcriptional up-regulation of fibronectin. J Pathol. (2014) 234:351–64. doi: 10.1002/path.2014.234.issue-3 25042645

[18] Wang Y Alla V Goody D Gupta SK Spitschak A Wolkenhauer O. Epigenetic factor EPC1 is a master regulator of DNA damage response by interacting with E2F1 to silence death and activate metastasis-related gene signatures. Nucleic Acids Res. (2016) 44:117–33. doi: 10.1093/nar/gkv885 PMC4705687 26350215

[19] Goody D Gupta SK Engelmann D Spitschak A Marquardt S Mikkat S. Drug repositioning inferred from E2F1-Coregulator interactions studies for the prevention and treatment of metastatic cancers. Theranostics. (2019) 9:1490. doi: 10.7150/thno.29546 30867845 PMC6401510

[20] Steder M Alla V Meier C Spitschak A Pahnke J Fürst K. DNp73 exerts function in metastasis initiation by disconnecting the inhibitory role of EPLIN on IGF1R-AKT/STAT3 signaling. Cancer Cell. (2013) 24:512–27. doi: 10.1016/j.ccr.2013.08.023 24135282

[21] Ai W Li H Song N Li L Chen H. Optimal method to stimulate cytokine production and its use in immunotoxicity assessment. Int J Environ Res Public Health. (2013) 10:3834–42. doi: 10.3390/ijerph10093834 PMC3799516 23985769

[22] Belo Y Mielko Z Nudelman H Afek A Ben-David O Shahar A. Unexpected implications of STAT3 acetylation revealed by genetic encoding of acetyl-lysine. Biochim Biophys Acta Gen Subj. (2019) 1863:1343–50. doi: 10.1016/j.bbagen.2019.05.019 PMC6625855 31170499

[23] Yan Y Zhang D Zhou P Li B Huang S-Y. HDOCK: a web server for protein-protein and protein-DNA/RNA docking based on a hybrid strategy. Nucleic Acids Res. (2017) 45:W365–73. doi: 10.1093/nar/gkx407 PMC5793843 28521030

[24] Vanommeslaeghe K Hatcher E Acharya C Kundu S Zhong S Shim J. CHARMM general force field: A force field for drug-like molecules compatible with the CHARMM all-atom additive biological force fields. J Comput Chem. (2010) 31:671–90. doi: 10.1002/jcc.21367 PMC2888302 19575467

[25] Mootha VK Lindgren CM Eriksson K-F Subramanian A Sihag S Lehar J. PGC-1alpha-responsive genes involved in oxidative phosphorylation are coordinately downregulated in human diabetes. Nat Genet. (2003) 34:267–73. doi: 10.1038/ng1180 12808457

[26] Subramanian A Tamayo P Mootha VK Mukherjee S Ebert BL Gillette MA. Gene set enrichment analysis: a knowledge-based approach for interpreting genome-wide expression profiles. Proc Natl Acad Sci. (2005) 102:15545–50. doi: 10.1073/pnas.0506580102 PMC1239896 16199517

[27] Liberzon A Birger C Thorvaldsdóttir H Ghandi M Mesirov JP Tamayo P. The Molecular Signatures Database (MSigDB) hallmark gene set collection. Cell Syst. (2015) 1:417–25. doi: 10.1016/j.cels.2015.12.004 PMC4707969 26771021

[28] Godec J Tan Y Liberzon A Tamayo P Bhattacharya S Butte AJ. Compendium of immune signatures identifies conserved and species-specific biology in response to inflammation. Immunity. (2016) 44:194–206. doi: 10.1016/j.immuni.2015.12.006 26795250 PMC5330663

[29] Da Huang W Sherman BT Lempicki RA. Systematic and integrative analysis of large gene lists using DAVID bioinformatics resources. Nat Protoc. (2009) 4:44–57. doi: 10.1038/nprot.2008.211 19131956

[30] Sherman BT Hao M Qiu J Jiao X Baseler MW Lane HC. DAVID: a web server for functional enrichment analysis and functional annotation of gene lists (2021 update). Nucleic Acids Res. (2022) 50:W216–21. doi: 10.1093/nar/gkac194 PMC9252805 35325185

[31] Li T Fu J Zeng Z Cohen D Li J Chen Q. TIMER2.0 for analysis of tumor-infiltrating immune cells. Nucleic Acids Res. (2020) 48:W509–14. doi: 10.1093/nar/gkaa407 PMC7319575 32442275

[32] Vera J Bachmann J Pfeifer AC Becker V Hormiga JA Darias NV. A systems biology approach to analyze amplification in the JAK2-STAT5 signaling pathway. BMC Syst Biol. (2008) 2:38. doi: 10.1186/1752-0509-2-38 18439261 PMC2386439

[33] Vera J Schmitz U Lai X Engelmann D Khan FM Wolkenhauer O. Kinetic modeling–based detection of genetic signatures that provide chemoresistance via the e2f1-p73/dnp73-mir-205 network. Cancer Res. (2013) 73:3511–24. doi: 10.1158/0008-5472.CAN-12-4095 23447575

[34] Xie D Pei Q Li J Wan X Ye T. Emerging role of E2F family in cancer stem cells. Front Oncol. (2021) 11:723137. doi: 10.3389/fonc.2021.723137 34476219 PMC8406691

[35] Zhao Z Cheng X Wang Y Han R Li L Xiang T. Metformin inhibits the IL-6-induced epithelial-mesenchymal transition and lung adenocarcinoma growth and metastasis. PLoS One. (2014) 9:e95884. doi: 10.1371/journal.pone.0095884 24789104 PMC4005743

[36] Yoon S Woo SU Kang JH Kim K Kwon M-H Park S. STAT3 transcriptional factor activated by reactive oxygen species induces IL6 in starvation-induced autophagy of cancer cells. Autophagy. (2010) 6:1125–38. doi: 10.4161/auto.6.8.13547 20930550

[37] Yoon J Grinchuk OV Tirado-Magallanes R Ngian ZK Tay EX Chuah YH. E2F and STAT3 provide transcriptional synergy for histone variant H2AZ activation to sustain glioblastoma chromatin accessibility and tumorigenicity. Cell Death Differ. (2022) 29:1379–94. doi: 10.1038/s41418-021-00926-5 PMC9287453 35058574

[38] Hutchins AP Diez D Takahashi Y Ahmad S Jauch R Tremblay ML. Distinct transcriptional regulatory modules underlie STAT3's cell type-independent and cell type-specific functions. Nucleic Acids Res. (2013) 41:2155–70. doi: 10.1093/nar/gks1300 PMC3575808 23295670

[39] Rasouli M Safari F. Principles of indirect co-culture method using transwell. Methods Mol Biol. (2024). doi: 10.1007/7651_2024_537 38502468

[40] Subauste CS Fuh F de Waal Malefyt R Remington JS. [amp]]alpha;β T cell response to toxoplasma gondii in previously unexposed individuals. J Immunol. (1998) 160:3403–11. doi: 10.4049/jimmunol.160.7.3403 9531300

[41] Simon S Labarriere N. PD-1 expression on tumor-specific T cells: Friend or foe for immunotherapy? Oncoimmunology. (2018) 7:e1364828. doi: 10.1080/2162402X.2017.1364828 PMC5739549 29296515

[42] Vivekanandan MM Adankwah E Aniagyei W Acheampong I Minadzi D Yeboah A. Impaired T-cell response to phytohemagglutinin (PHA) in tuberculosis patients is associated with high IL-6 plasma levels and normalizes early during anti-mycobacterial treatment. Infection. (2023) 51:1013–23. doi: 10.1007/s15010-023-01977-1 PMC10352402 36650358

[43] Hirano T. IL-6 in inflammation, autoimmunity and cancer. Int Immunol. (2021) 33:127–48. doi: 10.1093/intimm/dxaa078 PMC7799025 33337480

[44] Saifuddin M Spear GT Chang C Roebuck KA. Expression of MHC class II in T cells is associated with increased HIV-1 expression. Clin Exp Immunol. (2000) 121:324–31. doi: 10.1046/j.1365-2249.2000.01290.x PMC1905707 10931149

[45] Sun L Su Y Jiao A Wang X Zhang B. T cells in health and disease. Signal Transduct Target Ther. (2023) 8:235. doi: 10.1038/s41392-023-01471-y 37332039 PMC10277291

[46] Chang C-F D'Souza WN Ch'en IL Pages G Pouyssegur J Hedrick SM. Polar opposites: Erk direction of CD4 T cell subsets. J Immunol. (2012) 189:721–31. doi: 10.4049/jimmunol.1103015 PMC3392534 22675204

[47] Turan T Kongpachith S Halliwill K Roelands J Hendrickx W Marincola FM. A balance score between immune stimulatory and suppressive microenvironments identifies mediators of tumor immunity and predicts pan-cancer survival. Br J Cancer. (2021) 124:760–9. doi: 10.1038/s41416-020-01145-4 PMC7884411 33139798

[48] Seliger B Massa C. Immune therapy resistance and immune escape of tumors. Cancers. (2021) 13:551. doi: 10.3390/cancers13030551 33535559 PMC7867077

[49] van Allen EM Miao D Schilling B Shukla SA Blank C Zimmer L. Genomic correlates of response to CTLA-4 blockade in metastatic melanoma. Science. (2015) 350:207–11. doi: 10.1126/science.aad0095 PMC5054517 26359337

[50] Spranger S Bao R Gajewski TF. Melanoma-intrinsic β-catenin signaling prevents anti-tumor immunity. Nature. (2015) 523:231–5. doi: 10.1038/nature14404 25970248

[51] Mariathasan S Turley SJ Nickles D Castiglioni A Yuen K Wang Y. TGFβ attenuates tumor response to PD-L1 blockade by contributing to exclusion of T cells. Nature. (2018) 554:544–8. doi: 10.1038/nature25501 PMC6028240 29443960

[52] Dhanasekaran R Deutzmann A Mahauad-Fernandez WD Hansen AS Gouw AM Felsher DW. The MYC oncogene - the grand orchestrator of cancer growth and immune evasion. Nat Rev Clin Oncol. (2022) 19:23–36. doi: 10.1038/s41571-021-00549-2 34508258 PMC9083341

[53] Yang C Liu Y Hu Y Fang L Huang Z Cui H. Myc inhibition tips the immune balance to promote antitumor immunity. Cell Mol Immunol. (2022) 19:1030–41. doi: 10.1038/s41423-022-00898-7 PMC9424194 35962189

[54] Kornepati AV Vadlamudi RK Curiel TJ. Programmed death ligand 1 signals in cancer cells. Nat Rev Cancer. (2022) 22:174–89. doi: 10.1038/s41568-021-00431-4 PMC9989967 35031777

[55] Burkholder B Huang R-Y Burgess R Luo S Jones VS Zhang W. Tumor-induced perturbations of cytokines and immune cell networks. Biochim Biophys Acta. (2014) 1845:182–201. doi: 10.1016/j.bbcan.2014.01.004 24440852

[56] Andreu-Sanz D Kobold S. Role and potential of different T helper cell subsets in adoptive cell therapy. Cancers. (2023) 15:1650. doi: 10.3390/cancers15061650 36980536 PMC10046829

[57] Richter C Marquardt S Li F Spitschak A Murr N Edelhäuser BA. Rewiring E2F1 with classical NHEJ via APLF suppression promotes bladder cancer invasiveness. J Exp Clin Cancer Res. (2019) 38:1–16. doi: 10.1186/s13046-019-1286-9 31287003 PMC6615232

[58] Xu M Wang Y Xia R Wei Y Wei X. Role of the CCL2-CCR2 signaling axis in cancer: Mechanisms and therapeutic targeting. Cell Prolif. (2021) 54:e13115. doi: 10.1111/cpr.13115 34464477 PMC8488570

[59] Yoshimura T Li C Wang Y Matsukawa A. The chemokine monocyte chemoattractant protein-1/CCL2 is a promoter of breast cancer metastasis. Cell Mol Immunol. (2023) 20:714–38. doi: 10.1038/s41423-023-01013-0 PMC10310763 37208442

[60] Andrew DP Ruffing N Kim CH Miao W Heath H Li Y. C-C chemokine receptor 4 expression defines a major subset of circulating nonintestinal memory T cells of both Th1 and Th2 potential. J Immunol. (2001) 166:103–11. doi: 10.4049/jimmunol.166.1.103 11123282

[61] Jacobs JF Nierkens S Figdor CG de Vries IJ Adema GJ. Regulatory T cells in melanoma: the final hurdle towards effective immunotherapy? Lancet Oncol. (2012) 13:e32–42. doi: 10.1016/S1470-2045(11)70155-3 22225723

[62] Uciechowski P Dempke WC. Interleukin-6: A masterplayer in the cytokine network. Oncology. (2020) 98:131–7. doi: 10.1159/000505099 31958792

[63] Johnson DE O'Keefe RA Grandis JR. Targeting the IL-6/JAK/STAT3 signaling axis in cancer. Nat Rev Clin Oncol. (2018) 15:234–48. doi: 10.1038/nrclinonc.2018.8 PMC5858971 29405201

[64] Scheller J Chalaris A Schmidt-Arras D Rose-John S. The pro- and anti-inflammatory properties of the cytokine interleukin-6. Biochim Biophys Acta. (2011) 1813:878–88. doi: 10.1016/j.bbamcr.2011.01.034 21296109

[65] Dienz O Rincon M. The effects of IL-6 on CD4 T cell responses. Clin Immunol. (2009) 130:27–33. doi: 10.1016/j.clim.2008.08.018 18845487 PMC2660866

[66] Berraondo P Sanmamed MF Ochoa MC Etxeberria I Aznar MA Pérez-Gracia JL. Cytokines in clinical cancer immunotherapy. Br J Cancer. (2019) 120:6–15. doi: 10.1038/s41416-018-0328-y 30413827 PMC6325155

[67] Diehl S Rincón M. The two faces of IL-6 on Th1/Th2 differentiation. Mol Immunol. (2002) 39:531–6. doi: 10.1016/s0161-5890(02)00210-9 12431386

[68] Bent EH Millán-Barea LR Zhuang I Goulet DR Fröse J Hemann MT. Microenvironmental IL-6 inhibits anti-cancer immune responses generated by cytotoxic chemotherapy. Nat Commun. (2021) 12:6218. doi: 10.1038/s41467-021-26407-4 34711820 PMC8553783

[69] Huseni MA Wang L Klementowicz JE Yuen K Breart B Orr C. CD8+ T cell-intrinsic IL-6 signaling promotes resistance to anti-PD-L1 immunotherapy. Cell Rep Med. (2023) 4:100878. doi: 10.1016/j.xcrm.2022.100878 36599350 PMC9873827

[70] Briukhovetska D Dörr J Endres S Libby P Dinarello CA Kobold S. Interleukins in cancer: from biology to therapy. Nat Rev Cancer. (2021) 21:481–99. doi: 10.1038/s41568-021-00363-z PMC8173513 34083781

[71] Ozga AJ Chow MT Luster AD. Chemokines and the immune response to cancer. Immunity. (2021) 54:859–74. doi: 10.1016/j.immuni.2021.01.012 PMC8434759 33838745

[72] Heinzerling L Goldinger SM. A review of serious adverse effects under treatment with checkpoint inhibitors. Curr Opin Oncol. (2017) 29:136–44. doi: 10.1097/CCO.0000000000000358 28059853

[73] Zou S Tong Q Liu B Huang W Tian Y Fu X. Targeting STAT3 in cancer immunotherapy. Mol Cancer. (2020) 19:145. doi: 10.1186/s12943-020-01258-7 32972405 PMC7513516

[74] Dong J Cheng X-D Zhang W-D Qin J-J. Recent update on development of small-molecule STAT3 inhibitors for cancer therapy: from phosphorylation inhibition to protein degradation. J Med Chem. (2021) 64:8884–915. doi: 10.1021/acs.jmedchem.1c00629 34170703

