# Supplementary material for: E2F1-induced autocrine IL-6 inflammatory loop mediates cancer-immune crosstalk that predicts T cell phenotype switching and therapeutic responsiveness
Source: Front Immunol. 2024 Oct 31;15:1470368. doi: 10.3389/fimmu.2024.1470368 (PMC11560763; doi:10.3389/fimmu.2024.1470368)
Supplement: Supplementary file 1 [file DataSheet1.pdf]

# *Supplementary Material*

## 1 Supplementary Figure

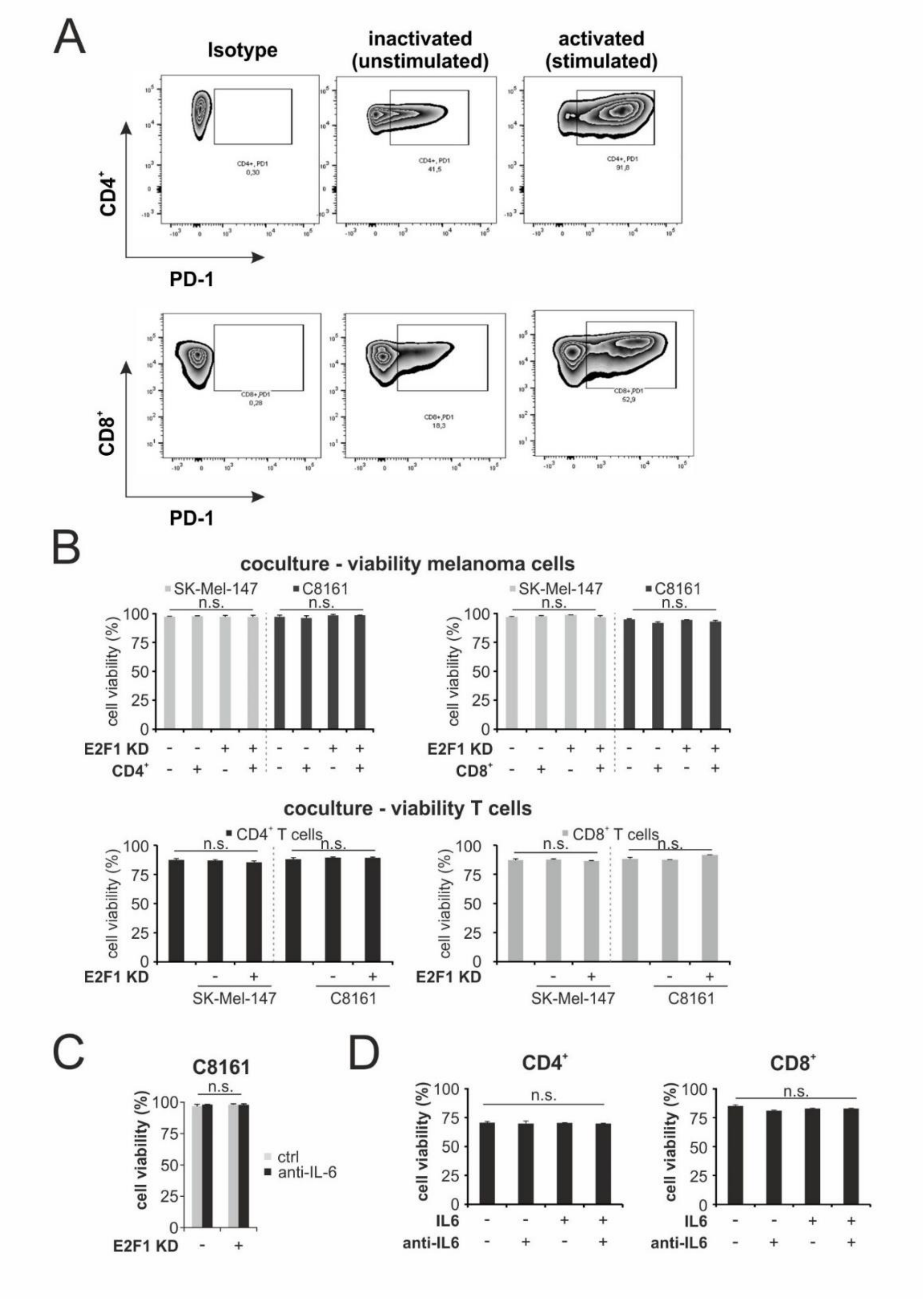


**Supplementary Figure S1.** Activity and viability of melanoma and selected T cells. **(A)** Flow cytometry analysis of PD1 expression in $CD4^+$ and $CD8^+$ T cells before and after PHA activation. **(B)**

Cell viability assays of cocultured melanoma and T cells. **(C, D)** Cell viability assays of melanoma **(C)** and T cells **(D)** in the presence or absence of anti-IL-6.
